# Impact of persistent barrier to gene flow and catastrophic events on red algae evolutionary history along the Chilean coast

**DOI:** 10.3389/fgene.2024.1336427

**Published:** 2024-03-08

**Authors:** Oscar R. Huanel, Alejandro E. Montecinos, Francisco Sepúlveda-Espinoza, Marie-Laure Guillemin

**Affiliations:** ^1^ Núcleo Milenio MASH, Facultad de Ciencias Biológicas, Pontificia Universidad Católica de Chile, Santiago, Chile; ^2^ IRL 3614 Evolutionary Biology and Ecology of Algae, Centre National de la Recherche Scientifique (CNRS), Sorbonne Université, Pontificia Universidad Católica de Chile, Universidad Austral de Chile, Station Biologique, Roscoff, France; ^3^ GEMA Center for Genomics, Ecology and Environment, Universidad Mayor, Santiago, Chile; ^4^ Instituto de Ciencias Ambientales y Evolutivas, Facultad de Ciencias, Universidad Austral de Chile, Valdivia, Chile; ^5^ Núcleo Milenio MASH, Instituto de Ciencias Ambientales y Evolutivas, Facultad de Ciencias, Universidad Austral de Chile, Valdivia, Chile; ^6^ Centro FONDAP de Investigación de Ecosistemas Marinos de Altas Latitudes (IDEAL), Valdivia, Chile

**Keywords:** comparative phylogeography, southeast pacific, rhodophyta, coastal uplift, actual and historical barriers to gene flow

## Abstract

Historical vicariance events, linked to the existence of stable physical barriers to gene flow, generate concordant genetic breaks in co-distributed species while stochastic processes (e.g., costal uplift) could cause species-specific genetic breaks as a result of local strong demographic bottlenecks or extinction. In Chile, previous studies show that the area of the 30°S-33°S could correspond to a stable barrier to gene flow that have affected the genetic structure of various algae and marine invertebrates. Here we sequenced two organellar genes (COI and rbcL) in four taxonomically accepted co-distributed red seaweeds species characterized by a low dispersal potential: *Mazzaella laminarioides, M. membranacea, Asterfilopsis disciplinalis, and Ahnfeltiopsis vermicularis*. Our results revealed the existence of ten strongly differentiated linages in the taxa studied. Strong genetic breaks, concordant in both space and time (divergence estimated to have occurred some 2.9–12.4 million years ago), were observed between taxa distributed across the 33°S. Conversely, in the Central/South part of the Chilean coast, the localization of the genetic breaks/sub-structure observed varied widely (36°S, 38°S, 39°S, and 40°S). These results suggest that a major historical vicariance event has modeled the genetic structure of several Chilean marine organisms in the north of the Chilean coast during the mid-Miocene, while more recent stochastic events and genetic drift could be the driving forces of genetic divergence/structuration in the central-southern part of the coast.

## 1 Introduction

Comparative phylogeography can be seen as a bridge linking the study of macro-scale biogeographic processes and local patterns of evolution at the within species level (Avise, 2000). The discipline has allowed to successfully evaluate the importance of historic and contemporary events in the evolutionary history of co-distributed species (Bermingham and Moritz, 1998; Arbogast and Kenagy, 2001; Bagley and Johnson, 2014). In the marine realm, main currents or strong upwelling systems can generate concordant genetic breaks in species of fishes, invertebrates, and algae (Kelly and Palumbi, 2010; Marko et al., 2010; Guillemin et al., 2016; Hiller and Lessios, 2020; Shelley et al., 2020). The imprint of oceanographic barriers on species genetic composition depends on both the type of barrier (i.e., hard or soft barriers; Cowman and Bellwood, 2013) and the species intrinsic characteristics. Species intrinsic characteristics, especially the ones directly related with the dispersal capacity, strongly affect the potential effect of soft barriers on genetic divergence (Kelly and Palumbi, 2010; Francisco et al., 2018; Ayres-Ostrock et al., 2019; Morales-González et al., 2019).

Other factors, as the rate of extinction and colonisation in the area under study (Fraser et al., 2018) or the population size (directly linked to the strength of genetic drift; Irwin, 2002; Kuo and Avise, 2005) can also affect a species response to a barrier to gene flow. These studies have corroborated the strong effect of drift on genetic differentiation, showing that species with small population size could be affected by very shallow and/or transitory barrier to gene flow. Stochastic events, as volcanic eruptions, floods, tsunami, or coastal uplift after earthquakes, generate demographic bottlenecks or even local extinctions (Castilla et al., 2010; Hsu et al., 2007; Pujolar et al., 2011; Schiel et al., 2019). These have lasting effects on population structure, especially in organisms with low dispersal capacity, but could be highly species-specific (Worthington Wilmer et al., 2011; Miura et al., 2017; Brante et al., 2019; Becheler et al., 2020; Parvizi et al., 2020). Indeed, the effect of drift and stochastic events could lead to lack of congruence in the spatial genetic structure of co-distributed species (Avise, 2000).

Comparative phylogeographic studies on marine organisms in the southeast Pacific mainly focused on vicariant processes at the origin of phylogenetic breaks concordant with major biogeographic barriers (Haye et al., 2014; Guillemin et al., 2016). The Chilean coast, spread over more than 4,500 km from 18°S to 56°S, is highly heterogeneous (variation in seawater temperature, salinity, upwelling regimes, and coastal topography between other) and encompasses two biogeographic breaks located at 30–33°S and 41–42°S (Camus, 2001; Thiel et al., 2007). The southern biogeographic break is related to the bifurcation of the Antarctic Circumpolar Current (ACC) and important seawater characteristics and topographic changes in the coastline occurring at 42°S has been proposed as important barriers to dispersal for marine organisms (Camus, 2001; Fernández et al., 2000). The biogeographic break at 30–33°S seems to be more permeable and taxa dependent (Santelices, 1980; Meneses and Santelices, 2000; Camus, 2001). For marine invertebrates and brown algae, the biogeographic break is reported at 30°S while for red algae the change in the community composition is observed at 32–33°S (Santelices, 1980; Meneses and Santelices, 2000; Camus, 2001). The 30–33°S area is an important transition zone in oceanographic features, with a weak but persistent upwelling in the north, and a strong but seasonal upwelling in the south (Broitman et al., 2001; Thiel et al., 2007; Tapia et al., 2014).

Numerous phylogeographic studies have corroborated the concordance between biogeographic and genetic breaks in Chilean marine invertebrates (Sanchez et al., 2011; Brante et al., 2012; Varela and Haye, 2012; Morales-González et al., 2019) and seaweeds (Tellier et al., 2009; Fraser et al., 2010; Macaya and Zucarello, 2010; Tellier et al., 2011; Montecinos et al., 2012). Along the coast located in between the two main biogeographic breaks, genetic breaks have been observed in some species. However, both the divergence time and the position of the genetic breaks seem to be more species specific (Montecinos et al., 2012; Haye et al., 2014; Guillemin et al., 2016; Morales-González et al., 2019; Quesada-Calderón et al., 2021). The coastline located between 36°S and 42°S has been regularly affected by earthquakes and coastal uplift that generated large but local mortality of intertidal organisms (Castilla, 1988; Castilla et al., 2010; Jaramillo et al., 2012; Hernández-Miranda et al., 2014; Brante et al., 2019). In addition, this area is characterized by environmental heterogeneity including variation in sea surface temperature and coastal marine currents (Atkinson et al., 2002) and large sandy beaches that can interrupt gene flow in organisms with low dispersal capacity (Fraser et al., 2010; Morales-González et al., 2019). It has been proposed that these local and transitory barriers to gene flow could have affected the genetic structure of species potentially strongly affected by genetic drift.

The present work aims to compare the genetic structure of the following four ecologically similar taxonomically accepted species of red algae: *M. laminarioides, M. membranacea*, *Asterfilopsis disciplinalis* (previously named *Ahnfeltiopsis furcellata* or *Gymnogongrus disciplinalis*, see Calderon and Boo, 2016), and *Ahnfeltiopsis vermicularis* (Calderon and Boo, 2017). *Ahnfeltiopsis vermicularis* has been historically reported in Chile under the names *Ahnfeltia durvillei* (Ramírez, 1991) and *Ahnfeltiopsis durvillei* (Hoffmann and Santelices, 1997), both names known as synonyms of *Gymnogongrus durvillei* (Guiry and Guiry, 2024). Molecular data associated with Chilean specimens collected under these names (Fredericq and Lopez-Bautista, 2002) appear as *Ahnfeltiopsis vermicularis* in the recent phylogeny of South American Phyllophoraceae published by Calderon and Boo (2017). These four species belong to the order Gigartinales, they are non-buoyant and present a limited dispersal capacity (Norton, 1992; Silva and DeCew, 1992; Hoffmann and Santelices, 1997). They are co-distributed along an extended part of the Chilean coast and are encountered in sympatry on rocky substrates located at high - mid intertidal (Santelices et al., 1989; Camus, 1992; Ramírez, 2010; Calderon and Boo, 2016). Previous studies in *M. laminarioides* have shown the existence of two strong genetic breaks in this species: one located at 33°S and concordant with a biogeographic break and one located at 37–39°S, in a part of the coast particularly affected by stochastic events such as earthquakes and coastal uplifts (Montecinos et al., 2012). Using sequences from two molecular markers, the mitochondrial COI and the cloroplastic *rbc*L, we tested for the existence of genetic breaks in three other species of red algae (i.e., *M. membranacea*, *As. disciplinalis*, and *Ah. vermicularis*) and searched for patterns of genetic divergence possibly congruent in time and space with the ones already observed in *M. laminarioides*. Our sampling cover part of the Chilean coast located between 28°55′S and 41°52′S and the algae were collected in 13 localities separated by around 100 km. The sampling includes the 30–33°S biogeographic break and the central-south part of the coastline characterized by mega-disturbance events and environmental heterogeneity. We hypothesized that all four co-distributed species of red algae will present congruent genetic breaks at 33°S, due to the effect of past vicariant events linked to the establishment of stable barrier to gene flow, while genetic divergence along the central-south part of the coastline, if detected, will be species specific. Because of the deep divergence within *M. laminarioides*, it has been proposed that the three genetic groups are incipient genetic species (Montecinos et al., 2012). We used species delimitation analyses to test if the genetic groups encountered could be considered as potentially new putative genetic species.

## 2 Materials and methods

### 2.1 Sampling

We sampled four species of red algae, *M. laminarioides* (N = 33)*, M. membranacea* (N = 152), As. *disciplinalis* (N = 176), and *Ah. vermicularis* (N = 157), when present, in 13 sampling sites along the Chilean coast. Sites ranged from Los Burros 28°55’S to Chiloe 41°52’S (Supplementary Table S1). For *M. laminarioides*, 16 individuals from Loberia (38°39’S-73°29’W) and 17 individuals from Nigue (39°17’S-73°13’W) were added to the 217 individuals previously sampled by Montecinos et al. (2012) (Supplementary Table S1). Samples were assigned to the four taxonomically accepted species using the external diagnostic characters described in Hoffmann and Santelices, (1997). All samples were stored in silica gel to ensure tissue preservation before DNA extraction.

### 2.2 DNA extraction, PCR amplification, sequencing, and alignment

For each sample, dry tissue was grounded in a MiniBeadBeater 24 (BioSpec Products, Inc. Bortlesville, United States) and DNA was isolated using the E. Z.N.A tissue DNA extraction kit following the provider instructions (Omega Biotek, Inc. Georgia, United States). A partial sequence of the mitochondrial Cytochrome c Oxidase I gen (COI) was amplified using the primers GazF1 and GazR1. Primers and PCR conditions were as described in Saunders, (2005). Additionally, a partial sequence of the chloroplast Ribulose bisphosphate carboxylase large chain gen (*rbc*L) was amplified for a subsampling of *M. laminarioides, M. membranacea*, As. *disciplinalis*, and *Ah. vermicularis*. The primers *rbc*L-F and *rbc*L-R (Hommersand et al., 1994) and the PCR conditions described in Fredericq and Lopez-Bautista (2002) were used for the *rbc*L amplification. PCR products were purified using the UltraClean^TM^ DNA Purification kit (MO BIO Laboratories, Carlsbad, CA, United States) and sequenced with an ABI Automatic Sequencer at the AUSTRAL-OMICS core-facility (Universidad Austral de Chile, Chile). Sequences were edited using Chromas v.2.33 (McCarthy, 1997) and aligned using Mega v.5 (Tamura et al., 2011).

In the case of *M. laminarioides*, 217 COI and 9 *rbc*L sequences already available in GenBank were added to our dataset (Montecinos et al., 2012).

### 2.3 Phylogenetic reconstruction

For the COI, 24 sequences of *Mazzaella* and 48 sequences of the family Phyllophoracea were downloaded from GenBank and added to our data set (Supplementary Table S2). In the same way, 33 sequences of *Mazzaella* and 83 sequences of the family Phyllophoracea were added to our *rbc*L data set (Supplementary Table S2). For both the COI and *rbc*L data sets, *Chondracanthus* and *Gigartina* sequences were used as outgroup for the *Mazzaella* phylogenetic reconstructions while *Mazzaella*, *Chondracanthus*, and *Chondrus* sequences were used as outgroup for the Asterfilopsis and *Ahnfeltiopsis* phylogenetic reconstructions (Supplementary Table S2). Duplicated haplotypes were removed from the data sets using DnaSP v6.12.03 prior to analyses (Rozas et al., 2017).

Phylogenetic reconstructions were performed independently for *Mazzaella,* Asterfilopsis, *and Ahnfeltiopsis* with the Maximum Likelihood (ML) method and Bayesian Inference (BI), using the COI and *rbc*L markers. ML analyses were performed using IQTree v1.6.12 (Trifinopoulos et al., 2016). The best-fit substitution model was selected using the Bayesian Information Criterion (BIC) implemented in IQTree v1.6.12 (Kalyaanamoorthy et al., 2017). The selected models for the COI were TIM3 + F + G4 for *Mazzaella* and TIM + F + I + G4 for Asterfilopsis *and Ahnfeltiopsis*. The selected models for the *rbc*L were TIM + F + I + G4 for *Mazzaella,* Asterfilopsis, and *Ahnfeltiopsis*. Statistical support was estimated using 1,000 ultrafast bootstrap replicates and the nonparametric SH-aLRT single branch test. BI analyses were performed using MrBayes v3.1.2 (Huelsenbeck and Ronquist, 2001). Four independent analyses were run. Four chains and five million of generations were used for each analysis. Trees and parameters were sampled every 1,000 generations and the default parameters were used to fit temperature and swapping. The first 25% of the sampled trees were discarded as burn-in to ensure stabilization. The remaining trees were used to compute a consensus topology and posterior probability values. Split frequencies (variance among the four independent runs) were below 0.003, confirming that the posterior probability distribution was accurately sampled.

### 2.4 Species delimitation analyses based on genetic data sets

To test the possible existence of cryptic putative genetic species within the four species sampled, we conducted three species delimitation analyses for *Mazzaella,* Asterfilopsis, and *Ahnfeltiopsis* using the COI and the *rbc*L datasets independently (see Supplementary Table S2 for more information about the list of sequences used in species delimitation analyses). First, the Automatic Barcode Gap Discovery (ABGD) was run online at http://wwwabi.snv.jussieu.fr/public/abgd/abgdweb.html. ABGD compare intraspecific and interspecific pairwise genetic distances to delimit species (Puillandre et al., 2012). We computed Kimura two-parameter (K2P) genetic distances and used default ABGD settings. Second, a General Mixed Yule Coalescent (GMYC) analysis was run. GMYC identifies a threshold value for the shift in branching rate from coalescent lineage branching to interspecific diversification on an ultrametric tree and explicitly delimits “independently evolving” clusters (i.e., putative species; Monaghan et al., 2009; Pons et al., 2006). Branch lengths were estimated under a relaxed log-normal clock using the Bayesian analysis implemented in BEAST v1.8.2 (Drummond et al., 2012). A coalescent (constant size) prior was used, and Markov Chains Monte Carlo (MCMC) were run for 20 million generations. Trees were sampled each 1,000 generations with a 10% burn-in. A visual inspection of MCMC progression was performed to corroborate stabilization using Tracer v1.8 (Drummond et al., 2012). An ultrametric tree was constructed using Tree Annotator v1.8 (Drummond et al., 2012). Since the multiple-thresholds approach tends to overestimate the number of delineated species (Fujisawa and Barraclough, 2013) only the single-threshold (Pons et al., 2006) versions of GMYC was fitted on the ultrametric tree using the SPLITS package for R.

The bPTP analysis was performed using a bifurcated phylogenetic input tree as implemented in bPTP web server (https://species.h-its.org; Zhang et al., 2013). The parameters were set as following: Markov chain Monte Carlo = 500,000 generations; thinning = 100; burn-in = 0.1; and the convergence were thoroughly checked to ensure that the result was reliable. The most plausible delimitations are reported with their associated Bayesian support values; higher Bayesian support value of a node indicates that all descendants from this node are more likely to be conspecific.

### 2.5 Divergence time estimation

Divergence time between putative genetic species of *Mazzaella,* Asterfilopsis, and *Ahnfeltiopsis* was estimated using BEAST v2.5 (Bouckaert et al., 2019) with a single concatenated matrix with two partitions, the COI and the *rbc*L. One sequence of each putative genetic species was used for the analysis, only putative genetic species for which both COI and the *rbc*L were available were used in the analysis (see Supplementary Table S2). A relaxed uncorrelated log-normal clock and Yule model were applied as tree priors for each partition. Substitution models GTR + I + G and GTR + I were applied for the COI and the *rbc*L, respectively. Best fit models were selected using BIC in jModelTest (Darriba et al., 2012). Three chains were run for 500 million generations and trees were sampled every 5,000 generations. Mutation rates of 0.350% (Hu et al., 2010) and 0.127% (Kamiya et al., 2004) were used for the COI and *rbc*L, respectively. Since the fossil record of Florideophyceae is exceptionally scarce (Yang et al., 2016), time of divergence between Gigartinaceae and Phyllophoraceae was calibrated at 113 Ma, as proposed in TimeTree (Kumar et al., 2022). Calibration point was set as normal distribution with 113 Ma as the mean and 5% of the mean as standard deviation. Convergence of model parameters was estimated by plotting the marginal posterior probabilities *versus* generations in Tracer v1.8 (Rambaut et al., 2018). Effective sample size values were estimated for each parameter to ensure adequate mixing of the MCMC (ESSs >200). The final consensus tree was produced using TreeAnnotator v1.8 (Bouckaert et al., 2019).

### 2.6 Genetic diversity estimates

The genetic diversity within each putative genetic species (see results for more details) and within each population of *Mazzaella,* Asterfilopsis, and *Ahnfeltiopsis* was evaluated using five indices: the number of haplotypes (nH), gene diversity (H), nucleotide diversity (π), number of polymorphic sites (S), and number of private haplotypes (Hpriv). These calculated for COI dataset in DNASP 4.10.3 (Rozas et al., 2003). As only a few haplotypes, at most, were detected within putative genetic species for the *rbc*L marker it was not used for genetic diversity analyses.

### 2.7 Phylogeographic structure

For *Mazzaella,* Asterfilopsis, and *Ahnfeltiopsis*, genealogical relationship between COI sequences were reconstructed using Median-Joining algorithm implemented in Network v5.0.1.1 (Bandelt et al., 1999). Population genetic structure was examined by pairwise Φ_ST_ between populations within each putative genetic species; and significance of the Φ_ST_ values was estimated using 1,000 permutations (Excoffier et al., 1992). For three putative genetic species (i.e., *M. membranacea*, *As. disciplinalis*, and *Ah*. *vermicularis*; see results section for more details), analyses of molecular variance (AMOVA) were conducted in Arlequin (Excoffier and Lischer, 2010) to detect spatial partitioning of genetic variance within sampling sites, among sampling sites within genetic groups and among genetic groups. Levels of significance were tested using 1,000 permutations (Excoffier and Lischer, 2010). As for genetic diversity analyses, the *rbc*L marker was not used for phylogeographic structure analyses.

### 2.8 Demographic history

For each putative genetic species of *Mazzaella*, *Asterfilopsis*, and *Ahnfeltiopsis,* three complementary approaches were used to infer their historical demography. Tajima’s D (Tajima, 1989) and Fu’s Fs (Fu, 1997) tests were used to assess significant excess of rare alleles, performing 10,000 bootstraps replicated using Arlequin v3.5 (Excoffier and Lischer, 2010). Moreover, the observed mismatch distribution was compared with expected values under a model of sudden demographic expansion (Rogers and Harpending, 1992), and statistical significance was tested with the sum of squared deviation (SSD) and the Harpending’s Raggedness index (Rag) after 10,000 bootstraps using Arlequin v3.5 (Excoffier and Lisher, 2010). Finally, Bayesian skyline plots (BSP) were constructed using BEAST v2.5 (Bouckaert et al., 2019). The best-fit substitution models, selected using BIC in IQTree v1.6.12 (Kalyaanamoorthy et al., 2017), were: TPM3 + F for *M. membranacea*, HKY + F for *As. disciplinalis*, HKY + F + I for *Ah. vermicularis* and HKY + F for *Ah*. sp. 2. Bayesian skyline plots were constructed using 100 million iterations. Posterior distribution of parameters was approximated by Markov Chain Monte Carlo (MCMC) sampled every 10,000 iterations after a discarded burn in of five million iterations. A relaxed log-normal molecular clock was used, with a substitution rate of 0.35% per million years estimated for COI (Hu et al., 2010) to test for historical changes of effective population size. BSP reconstructions were visualized in Tracer v1.8 (Rambaut et al., 2018).

## 3 Results

A partial COI segment of 572 pb was successfully sequenced in 250 *M. laminarioides,* 152 *M. membranacea,* 176 As. *disciplinalis*, and 157 *Ah. vermicularis*. Partial *rbc*L sequences of 919 pb were sequenced for nine, four, eleven and eight individuals of *M. laminarioides, M. membranacea,* As. *disciplinalis*, and *Ah. vermicularis*, respectively.

### 3.1 Phylogeny and species delimitation

**FIGURE 1:**
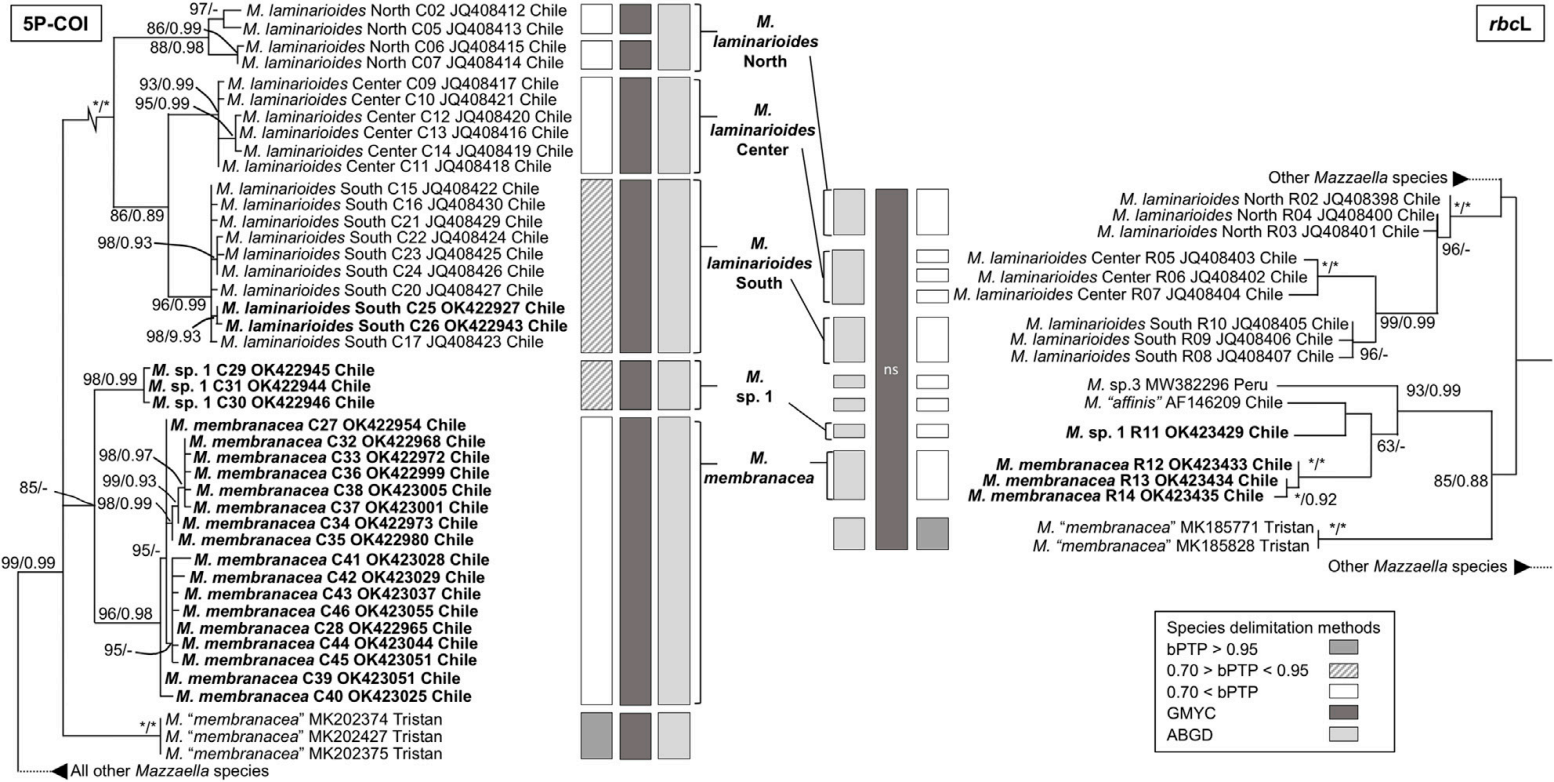
Maximun likelihooh phylogram of *Mazzaella* species inferred from COI and rbc*L* sequences. ML bootstrap values (≥60) and BI posterior probabilities (≥90) are indicated near branches; asterisk (*) indicates full support. Sequences obtained in our study are in bold. The results of three species delimitation methods (bPTP, GMYC, ABGD; see material and methods for more details) are indicated for both the COI and the rbc*L* markers. For the GYMC species delimitation method, analyses for which the model considering the existence of distinct species was not statistically significantly better than the null model (i.e., leading to the lumping of all sequences used) are indicated by “ns”.

**FIGURE 2:**
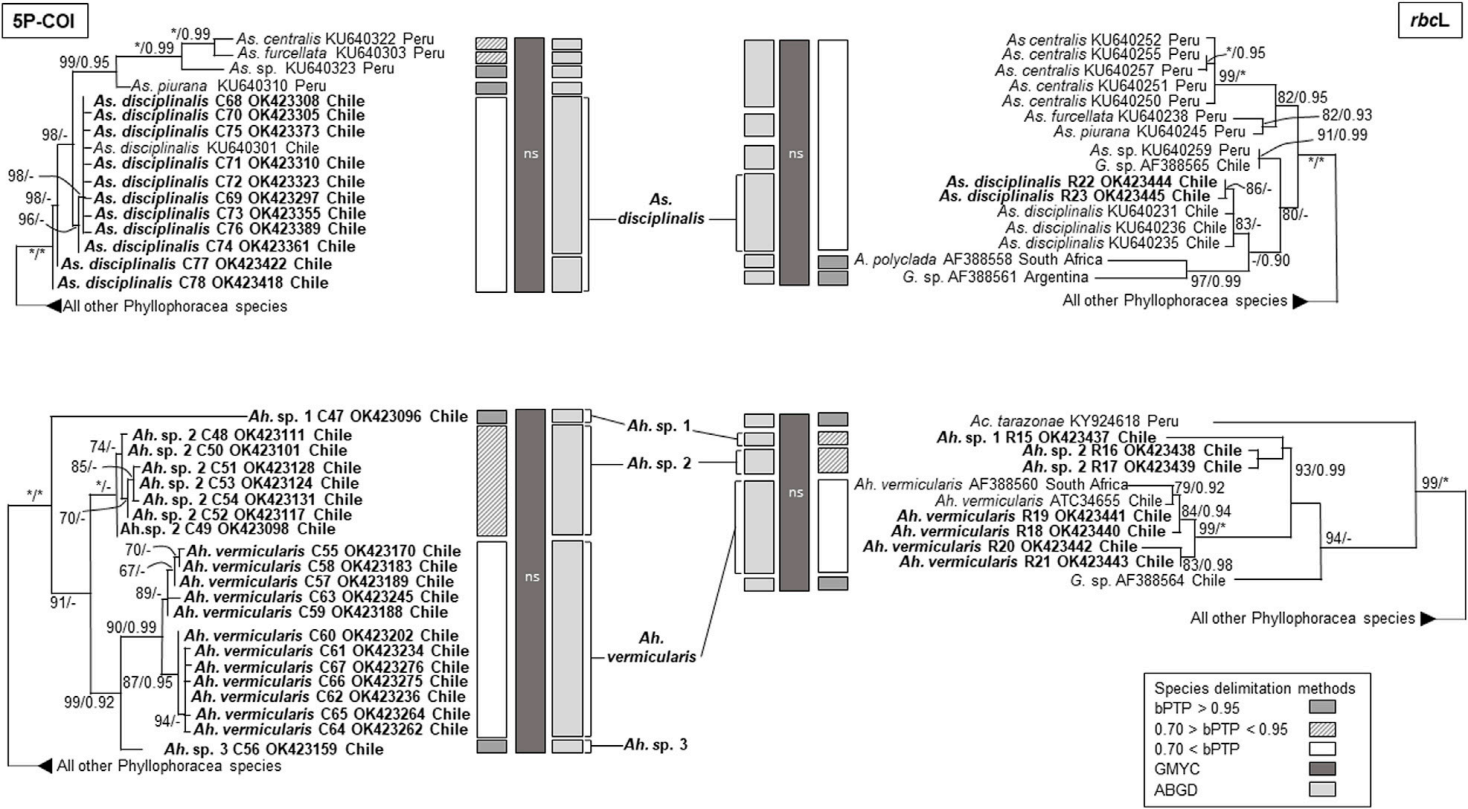
Maximun likelihooh phylogram of *Asterfilopsis* and *Ahnfeltiopsis* species inferred from COI and rbc*L* sequences. ML bootstrap values (≥60) and BI posterior probabilities (≥90) are indicated near branches; asterisk (*) indicates full support. Sequences obtained in our study are in bold. The results of three species delimitation methods (bPTP, GMYC, ABGD; see material and methods for more details) are indicated for both the COI and the rbc*L* marker. For the GYMC species delimitation method, analyses for which the model considering the existence of distinct species was not statistically significantly better than the null model (i.e., leading to the lumping of all sequences used) are indicated by “ns”.

The phylogenetic analyses based on COI and *rbc*L sequences from Chile formed distinct well supported monophyletic lineages within all four species, except for As. *disciplinalis*, for which all *rbc*L sequences from Chile form a unique discrete monophyletic lineage while the COI sequences from Chile form a paraphyletic group (Figures 1, 2). Both the COI and *rbc*L separate *M. laminarioides* into three putative genetic species (*M. laminarioides* North, *M. laminarioides* Center, and *M. laminarioides* South; names follow Montecinos et al., 2012; Figure 1) and *M. membranacea* into two putative genetic species (*M. membranacea* and *M.* sp. 1; Figure 1). For the *rbc*L marker, all sequences of *M. membranacea* and *M.* sp. 1 from Chile were closely related to *M.* “*affinis*” and *M.* sp. 3 from Chile and Peru, respectively (Figure 1). COI and *rbc*L sequences of individuals identified as *M. “membranacea*” and collected in Tristan Island (South Atlantic) appear as sister clade to the sequences obtained from Peru and Chile (Figure 1). For the COI, haplotypes C77 and C78 of *As. disciplinalis* were retrieved as a basal polytomy in respect to the rest of the sequences obtained (Figure 2). Individuals collected under the name *Ahnfeltiopsis vermicularis* were separated in four well defined genetic lineages for the COI; *Ah. vermicularis, Ah.* sp. 1, *Ah.* sp. 2, and *Ah.* sp. 3 (Figure 2). *Ahnfeltiopsis vermicularis* (including the sequence AF388560 from South Africa), *Ah.* sp. 1 and *Ah.* sp. 2 also appear as distinct lineages in the *rbc*L phylogenetic tree; *rbc*L fragment of *Ah.* sp. 3 could not be amplified. All *Asterfilopsis* and *Ahnfeltiopsis* sequences obtained in our study clustered with close by Phyllophoracea species from the Southern Hemisphere (*Asterfilopsis*: with four newly described species of *Asterfilopsis* from Peru*; Ahnfeltiopsis*: with *Acletoa tarazonae* KY924618 from Peru and *Gymnogongrus* sp. AF388564 from Chile; Calderon and Boo, 2016; Calderon and Boo, 2017; Figure 2).

In congruence with the well supported lineages detected in the tree reconstructions, ABGD results for both the COI and the *rbc*L markers suggest the existence of five putative genetic species in our *Mazzaella* data set: *M. laminarioides* North, *M. laminarioides* Center, *M. laminarioides* South, *M. membranacea*, and *M.* sp. 1 (Figure 1). For the COI, the single-threshold GMYC model (L_GMY_ = 294.241 > L_0_ = 289.290, *p* < 0.005) was mostly congruent, but separated *M. laminarioides* North in two clusters (Figure 1). The GMYC analysis of the *rbc*L phylogeny could not reject the null hypothesis of a single species (L_GMY_ = 32.544 > L_0_ = 32.083, *p* = 0.63; Figure 1). In *Mazzaella*, the separation of *M. “membranacea*” from Tristan Island was sustained by all three species delineation methods for the COI and by two of the three methods used for the *rbc*L (Figure 1). For *Asterfilopsis*, the ABGD analysis using sequences of COI support the separation of the haplotypes C77 and C78 from the rest sequences of *As. disciplinalis* obtained in Chile (including *As. disciplinalis* KU640301 from Chile sequenced by Calderon and Boo, 2016; Figure 2). This result was not supported by the GMYC analyses (L_GMY_ = 94.777 > L_0_ = 94.025, *p* = 0.47) nor the ones realized using the bPTP method (Figure 2). For the *rbc*L, only the ABGD analysis was able to clearly separate recently diverged species from Chile and Peru and grouped all *As. disciplinalis* sequences from Chile (i.e., the ones obtained in our study and the ones already published in Calderon and Boo, 2016; Figure 2). For *Ahnfeltiopsis*, the ABGD analyses for the COI and *rbc*L are congruent and support the existence of four putative genetic species in our data set: *Ah. vermicularis*, *Ah.* sp. 1, *Ah.* sp. 2, and *Ah.* sp. 3 (*rbc*L sequence of *Ah.* sp. 3 not available for the analysis; Figure 2). The bPTP analyses support the existence of these putative genetic species (Figure 2). The GMYC analyses for the COI and the *rbc*L markers could not reject the null hypothesis of a single species (L_GMY_ = 137.733 > L_0_ = 135.077, *p* = 0.07, and L_GMY_ = 53.266 > L_0_ = 52.813, *p* = 0.63, for COI and *rbc*L, respectively; Figure 2).

### 3.2 Divergence times

**FIGURE 3:**
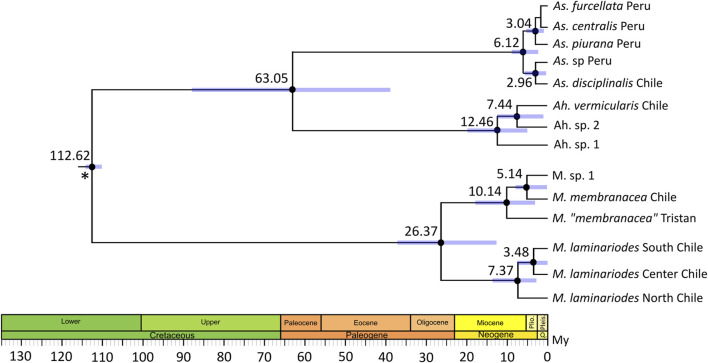
Divergence time estimates using Bayesian relaxed molecular clock (BEAST) inferred from a two-partitioned nucleotide matrix. A 572 bp COI segment and a 919bp *rbc*L segment were used. Black dots indicate 90% relevant nodes. Blue bars show 95% credibility intervals for node ages. Scale axis is in millions of years (My). Asterisk represent calibration point, time of divergence between Gigartinaceae and Phyllophoraceae was set to 113 My (Kumar et al., 2022). Accession numbers of the sequences used in the time-calibrated tree analysis are listed in Supplementary Table S2.

The chronogram reconstructed with BEAST is shown in Figure 3; node age estimations exhibited relatively large confidence intervals. According to this, the separation between *M. laminarioides* North and *M. laminarioides* Center/South occurred 7.37 Ma (95% Credibility Intervals, CI: 13.22–1.82 Ma), while the separation between *M. laminarioides* Center and *M. laminarioides* South occurred 3.48 Ma (CI: 6.84–0.01 Ma). For *M. membranacea* and *M.* sp. 1, the divergence times was estimated to have occurred at 5.14 Ma (CI: 9.12–0.06 Ma). *Mazzaella “membranacea*” collected in Tristan Island and *M. membranacea* from Chile and *M.* sp. 1 were differentiated around 10.14 Ma (CI: 16.03–4.31 Ma). For *Ahnfeltiopsis*, the separation between *Ah.* sp. 2 and *Ah. vermicularis* was dated at approximately 7.44 (CI: 12.59–0.77 Ma) and the separation between *Ah.* sp. 1 and *Ah.* sp. 2/*Ah. vermicularis* at approximately 12.46 Ma (CI: 20.19–5.39 Ma). This last divergence time could be biased since *Ah.* sp. 3 was not included in the analysis (i.e., no *rbc*L sequence available).

### 3.3 Diversity and distribution of putative genetic species

**FIGURE 4:**
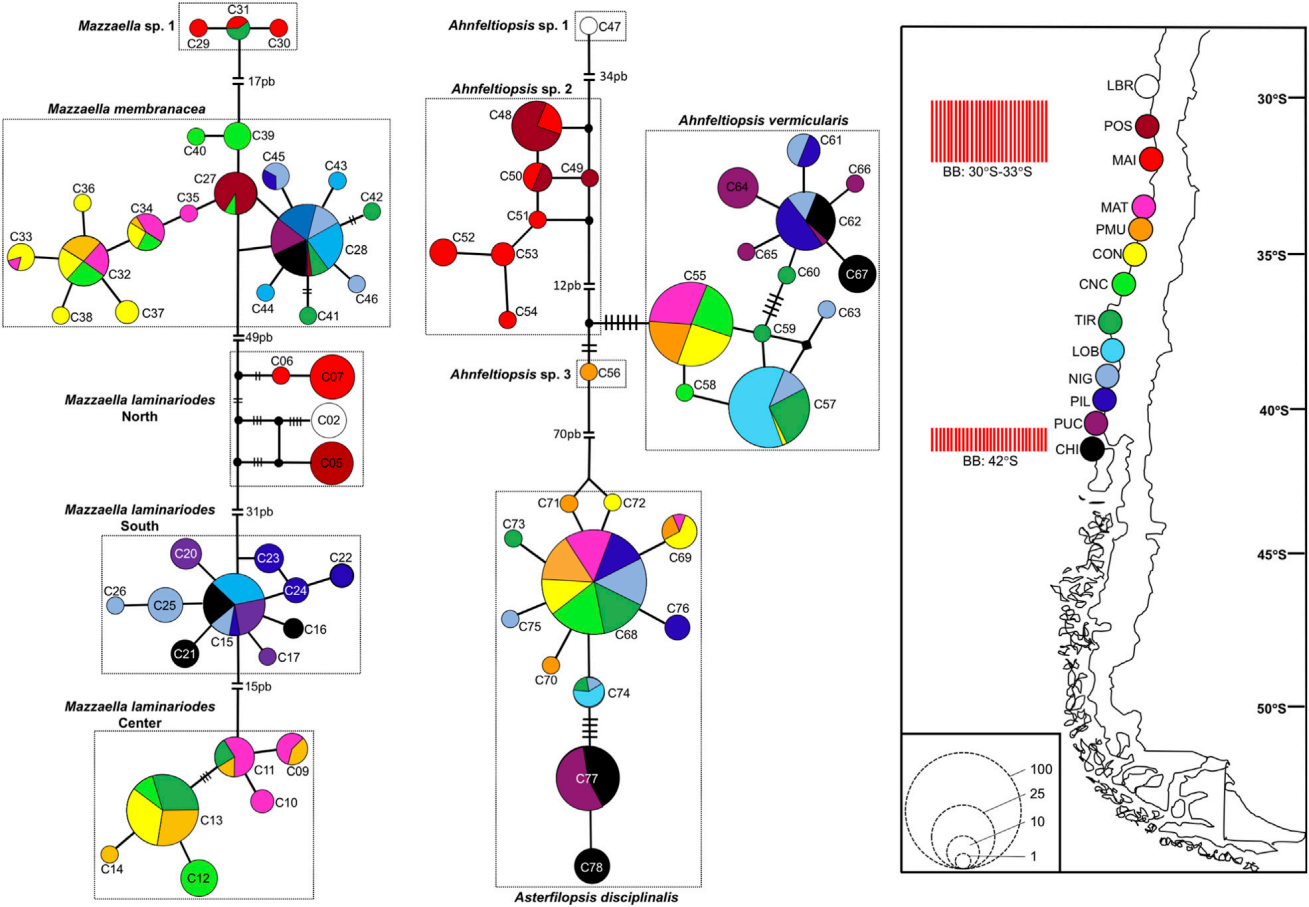
Haplotype network inferred from COI sequences in *Mazzaella*, *Asterfilopsis*, and *Ahnfeltiopsis* Chilean species. Map show the sampling sites and the two biogeographic breaks (BB red bars) described for the Chilean coast (Camus, 2001; Thiel et al., 2007). In the networks, each circle represents a haplotype. Circle size is proportional to the haplotype frequency while colors correspond to sampling sites where the haplotype was observed. Black circles represent hypothetical un-sampled haplotypes. For haplotypes separated by more than one mutational step, the number of steps is indicated by black bars (for values <7), or by the number of base pairs, bp (for valuer ≥7). Haplotype number as in Supplementary Table S3. Population abbreviations: Los Burros (LBR), Puerto Oscuro (POS), Maitencillo (MAI), Matanza (MAT), Pichilemu (PMU), Constitución (CON), Concepción (CNC), Tirua (TIR), Loberia (LOB), Nigue (NIG), Pilolcura (PIL), Pucatrihue (PUC), Chiloé (CHI); more details in Supplementary Table S1.

*Mazzaella laminarioides* North, Center, and South are well separated along the Chilean coast with *M. laminarioides* North distributed between the sampling sites LBR and MAI (28°55’S-32°37’S), *M. laminarioides* Center between the sampling sites MAT and TIR (34°05’S-37°38’S), and *M. laminarioides* South between the sampling sites LOB and CHI (38°39′S-41°52’S) (Figure 4; Supplementary Table S1). *Mazzaella laminarioides* South has been reported previously as spanning all the way down to Tierra del Fuego (∼54°S; Montecinos et al., 2012; southern sites not included in the present study). *Mazzaella membranacea* is a species presenting a large distribution in Chile, with individuals sampled from ∼31°S down to ∼41°S (from POS to CHI, except in MAI; Figure 4; Supplementary Table S1). The species has been mostly reported in the center south of Chile (Ramírez, 1991), where our samples have been taken; but some reports exist for Tierra del Fuego (Ramírez, 2010). Contrastingly, *M.* sp. 1 was encountered only in two sampling sites: MAI and TIR (32°37’S and 37°38’S, respectively; Figure 4; Supplementary Table S1). *Asterfilopsis disciplinalis* was encountered from MAT to CHI (34°05’S-41°52’S; Figure 4; Supplementary Table S1). Three of the four putative genetic species of *Ahnfeltiopsis* are fairly well separated along the Chilean coast with *Ah.* sp. 1 present only in LBR (28°55’S), *Ah.* sp. 2 in POS and MAI (32°37’S-32°37’S) and *Ah. vermicularis* from MAT to CHI (34°05’S-41°52’S) (Figure 4; Supplementary Table S1). *Ahnfeltiopsis vermicularis* extend to the coasts of South Africa (AF388560: Hondeklip Bay, Northern Cape Peninsula; Figure 2). The only sample named *Ahnfeltiopsis* sp. 3 was located in PMU (34°23’S; Figure 4; Supplementary Table S1).

Results reported in Table 1 show that all six commonly sampled putative genetic species show a high genetic diversity. Fairly similar and high values of gene diversity were reported for these species, while nucleotide diversity was more variable. For *M. laminarioides* North, *M. laminarioides* Center, *M. laminarioides* South, *M. membranacea, As*. *disciplinalis* and *Ah*. *vermicularis* values obtained for H varied between 0.56 and 0.78 while values obtained for π varied between 0.0021 and 0.0103 (Table 1). In *Ah*. sp. 2, a putative genetic species restricted to POS and MAI, estimates of genetic diversity (36 individuals sequenced; H = 0.61; π = 0.0022; Table 1) were fairly similar to the ones obtained for the more widely distributed genetic species. For *M.* sp. 1, a putative genetic species for which only nine individuals were sequenced, a low level of genetic diversity was observed (H = 0.41; π = 0.0008; Table 1). In four of the six more widely distributed genetic species no clear geographic pattern of changes in genetic diversity were observed along the Chilean coast (i.e., *M. laminarioides* North, *M. laminarioides* Center, *M. laminarioides* South and *As*. *disciplinalis*; Supplementary Table S1). However, in *M. membranacea* the most southern sites of PUC and CHI were less genetically diverse (H = 0.00; π = 0.00) than the northernmost sites located from POS to PIL (0.13 < H < 0.76; 0.0002 < π < 0.0039), while in *Ahfeltiopsis vermicularis* the genetic diversity of the northern part of the species distribution seems to be slightly lower than the one observed in the south (MAT-LOB: 0.00 < H < 0.26; 0.00 < π < 0.0014; NIG-CHI: 0.34 < H < 0.76; 0.0006 < π < 0.0089; Supplementary Table S2).

**TABLE 1:** Indices of genetic diversity and test of historical demographic change based on COI sequences obtained for each *Mazzaella*, *Asterfilopsis*, and *Ahnfeltiopsis* species sampled along the Chilean coast.

		Genetic diversity indices				Tests of demographic changes			
Species	N	nH	H^a^	π (0.10^–2^)^a^	S	Tajima′s D (*p*-value)	Fu′s Fs (*p*-value)	SSD (*p*-value)^b^	Rag (*p*-value)^b^
*Mazzaella*									
*M. laminarioides* North	52	4	0.68 (0.03)	1.03 (0.03)	13	†	†	†	†
*M. laminarioides* Center	104	6	0.61 (0.05)	0.30 (0.03)	7	†	†	†	†
*M. laminarioides* South	94	10	0.73 (0.02)	0.21 (0.02)	9	†	†	†	†
*M. membranacea*	143	17	0.78 (0.03)	0.40 (0.02)	19	−0.94 (0.18)	−4.49 (0.06)	0.02 (0.49)	0.04 (0.60)
*M.* sp. 1	9	3	0.41 (0.03)	0.08 (0.04)	2	-	-	-	-
*Asterfilopsis*									
*As. disciplinalis*	140	11	0.56 (0.04)	0.36 (0.04)	13	−0.33 (0.40)	−0.86 (0.43)	0.08 (0.01)	0.17 (0.94)
*Ahnfeltiopsis*									
*Ah. vermicularis*	155	12	0.75 (0.02)	0.600.03)	11	−0.08 (0.60)	0.72 (0.68)	0.09 (0.02)	0.20 (<0.01)
*Ah.* sp. 1	1	1	-	-	-	-	-	-	-
*Ah.* sp. 2	36	7	0.61 (0.09)	0.22 (0.04)	5	0.13 (0.60)	−1.63 (0.16)	0.01 (0.65)	0.07 (0.64)
*Ah.* sp. 3	1	1	-	-	-	-	-	-	-

N, total number of sequences; nH, number of haplotypes; H, gene diversity; π, nucleotide diversity, and S: number of polymorphic sites. Reported are Tajima’s D, Fu’s Fs, goodness of fit for a model of population expansion calculated from the sum of squared deviation (SSD), and Ramos-Onsins and Rozas’ *R* ^2^ tests, each followed by its respective *p*-value.

^a^ Standard deviations are in brackets.

^b^ *p*-value >0.05 means that the null hypothesis of sudden expansion cannot be rejected.

†: value previously estimated using more populations, especially for *Mazzaella laminarioides* South, in Montecinos et al. (2012).

-: not estimated because of small sample size.

### 3.4 Existence of substructure in species presenting a large distribution in Chile

For *M. laminarioides* Center and South, no clear genetic structuring was observed, with common haplotypes shared among various sampling sites (C11 and C13 for *M. laminarioides* Center and C15 for *M. laminarioides* South; Figure 4). Contrastingly, in *M. laminarioides* North no haplotypes were shared among sampling sites (Figure 4; Supplementary Table S3). These haplotypes were separated by at least nine mutations (Figure 4), leading to a strong genetic substructure (0.97 < Φ_ST_ values calculated among localities <1.00; Supplementary Table S4). In *M. membranacea,* except for one sample from POS for which the most common haplotype sequenced in the southern part of the species distribution was observed (haplotype C28; Figure 4), haplotypes sequenced in the northern part of the species distribution (i.e., from POS to CNC; 31°24’S-36°31'S) where distinct than the ones from the south (i.e., TIR to CHI; 37°38’S-41°52'S). Only one mutation separated C28, sampled in all six southernmost populations, from C27 encountered in CNC and POS (Figure 4; Supplementary Table S3). Pairwise Φ_ST_ calculated among localities located between POS and CNC ranged from 0.03 to 0.88 (Φ_ST_ = 0.41 ± 0.31) and the one calculated among localities located between TIR and CHI ranged from 0.00 to 0.14 (Φ_ST_ = 0.06 ± 0.05) (Table 2; Supplementary Table S4). Pairwise Φ_ST_ calculated among localities from the two distinct genetic groups ranged from 0.47 to 0.91 (Φ_ST_ = 0.78 ± 0.13; Table 2; Supplementary Table S4). The results from AMOVA indicated that the majority of variation was observed between genetic groups (i.e., 62%; Table 3). *Mazzaella* sp. 1 did not present any sign of genetic structure, with the haplotype C31 shared between MAI and TIR (Figure 4; Supplementary Table S3).

**TABLE 2:** Pairwise Φ_ST_ between sampling sites estimated from COI data set in *Mazzaella membranacea*, *Asterfilopsis disciplinalis*, and *Ahnfeltiopsis vermicularis*. Sampling sites included in each genetic group are indicated between square brackets.

	Within the genetic group corresponding to the northern part of the species distribution	Within the genetic group corresponding to the southern part of the species distribution	Between genetic groups
*M. membranacea*	[POS, MAT, PMU, CON, CNC]	[TIR, LOB, NIG, PIL, PUC, CHI]	
Min - Max	0.032–0.887	0.000–0.149	0.477–0.917
Mean (SD)	0.412 (0.317)	0.064 (0.051)	0.780 (0.132)
*As. disciplinalis*	[MAT, PMU, CON, CNC, TIR, LOB, NIG, PIL]	[PUC, CHI]	
Min–Max	0.000–1.000		0.908–1.000
Mean (SD)	0.246 (0.334)	0.257	0.951 (0.030)
*Ah. vermicularis*	[MAT, PMU, CON, CNC, TIR, LOB]	[NIG, PIL, PUC, CHI]	
Min–Max	0.000–1.000	0.359–0.622	0.394–0.982
Mean (SD)	0.475 (0.455)	0.480 (0.097)	0.850 (0.177)

**TABLE 3:** Analysis of Molecular Variance (AMOVA) implemented to explore the spatial partitioning of variance within sampling sites, among sampling sites within genetic groups and among genetic groups in *Mazzaella membranacea*, *Asterfilopsis disciplinalis,* and *Ahnfeltiopsis vermicularis*.

Source of variation	d.f	Sum of squares	Variance components	Percentage of variation	*p*-value	SD +/−
*M. membranacea*						
Among genetic groups	1	78.021	1.034	61.94	0.000	0.000
Among sampling sites within genetic groups	9	35.284	0.276	16.57	0.000	0.000
Among individuals within sampling sites	132	47.360	0.358	21.49	0.000	0.000
Total	142	160.664	1.669			
*As. disciplinalis*						
Among genetic groups	1	119.291	2.455	93.27	0.000	0.000
Among sampling sites within genetic groups	8	5.524	0.040	1.54	0.000	0.000
Among individuals within sampling sites	130	17.771	0.136	5.19	0.019	0.003
Total	139	142.586	2.632			
*Ah. vermicularis*						
Among genetic groups	1	156.090	2.075	71.81	0.000	0.000
Among sampling sites within genetic groups	8	68.043	0.539	18.68	0.000	0.000
Among individuals within sampling sites	145	39.867	0.274	9.51	0.002	0.001
Total	154	264.000	2.890			

d.f.: degree of freedom; SD: +/−, standard deviation, levels of significance were tested using 1,000 permutations.

*Asterfilopsis disciplinalis* showed a clear genetic break between localities of PIL (39°4’S) and PUC (40°32'S), with four fixed mutations separating haplotypes from the northern and southern part of the species distribution (Figure 4). Pairwise Φ_ST_ calculated among localities from the two distinct genetic groups (0.91 < Φ_ST_ < 1.00; Supplementary Table S4) were higher than the ones calculated among localities located within each group (0.00 < Φ_ST_ < 1.00; Supplementary Table S4). The AMOVA shows that the largest fraction of the variation is distributed between genetic groups (i.e., 93%; Table 3), supporting the high level of genetic differentiation between the two genetic groups (Φ_ST_ = 0.95 ± 0.03; Table 2). The COI sequences obtained in Calderon and Boo, (2016) from Pichilemu (GenBank accession number KU640301) and Valdivia (GenBank accession number KU640298), both from sites located north of the 39°S genetic break, support our results. KU640301 correspond to the haplotype C68 and KU640298 to a new rare haplotype separated only by one mutation from C68.

In *Ah. vermicularis,* haplotypes encountered in the northern part of the species distribution (from MAT to LOB; 34°05’S-38°39'S) were separated from the ones sampled in the south (i.e., from NIG to CHI; 39°17’S-41°52'S) by one mutation (Figure 4). The pairwise Φ_ST_ values calculated among localities located between MAT and LOB ranged from 0.00 to 1.00 (Φ_ST_ = 0.47 ± 0.45) and the one calculated among localities located between NIG and CHI ranged from 0.35 to 0.62 (Φ_ST_ = 0.48 ± 0.09) (Table 2; Supplementary Table S4). The Φ_ST_ calculated among localities from the two distinct genetic groups ranged from 0.39 to 0.98 (Φ_ST_ = 0.85 ± 0.17) (Table 2; Supplementary Table S4). The AMOVA shows that the largest fraction of the variation is distributed between genetic groups (i.e., 72%; Table 3). *Ahnfeltiopsis* sp. 2 showed a reticulated network with two shared haplotypes between POS and MAI (haplotypes C48 and C50; Figure 4) and the Φ_ST_ calculated between these two sites was of 0.34 (Supplementary Table S4).

### 3.5 Historical demography

For *M. laminarioides*, tests of historical demographic change have already been reported for the three putative genetic species in Montecinos et al. (2012). This study revealed that *M. laminarioides* North had passed through a recent bottleneck while signs of population growth were detected for *M. laminarioides* South, linked with rapid post-glacial expansion. *Mazzaella laminarioides* Center did not reveal any clear pattern of effective population size fluctuations throughout time.

**FIGURE 5:**
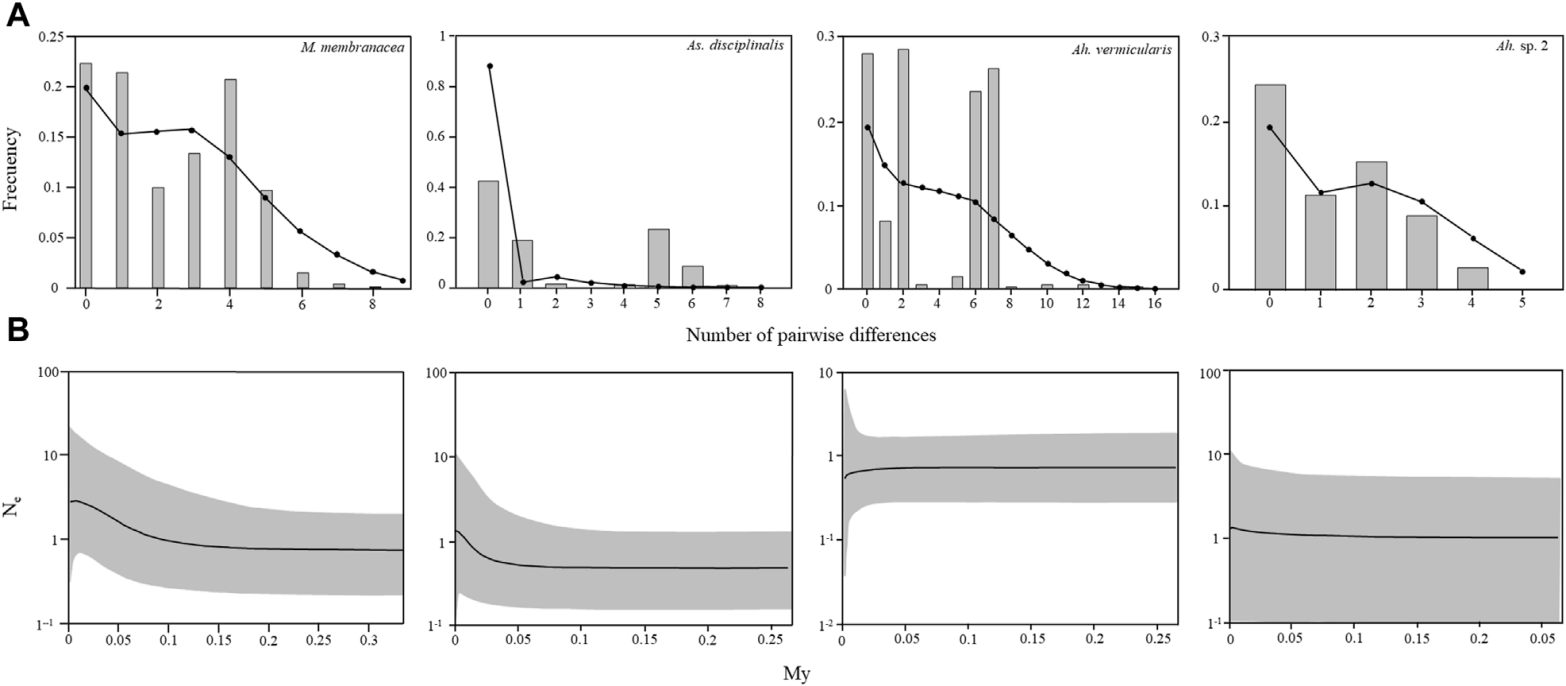
Mismatch distribution and Bayesian Skyline plots calculated with COI sequences for *Mazzaella membranacea, Asterfilopsis disciplinalis*, *Ahnfeltiopsis vermicularis*, and *Ah.* sp. 2. **(A)** Observed mismatch distribution, represented by grey bars, and expected mismatch distribution under a spatial expansion model represented by black circles. **(B)** Changes in effective population size estimated by Bayesian Skyline plots. Solid lines are the median posterior effective population size through time; gray area indicates the 95% confidence interval for each estimate.

For *M. membranacea, As. disciplinalis, Ah. vermicularis*, and *Ah.* sp. 2, the mismatch distributions are bimodal, with SSD and Rag index that generally did not allow to reject the hypothesis of a sudden expansion except for the SSD in the case of *As. disciplinalis* and both the SSD and the Rag index in the case of *Ah. vermicularis* (Table 1; Figure 5). Non-significant values were obtained for the Fu’s Fs and Tajima’s D tests for the four putative genetic species (Table 1). For *Ah. vermicularis* and *Ah.* sp. 2, BSP showed no evidence of clear demographic changes (Figure 5). Contrastingly, in *M. membranacea* and *As. disciplinalis* recent demographic increase are detected in the BSP analyses (more or less 25,000 years ago, Figure 5). These results are supported by fairly high negative values of Fu’s Fs and Tajima’s D in the case of these two putative genetic species (Table 1). However, only mild increase in Ne (i.e., effective population size), at most, were detected in the BSP.

## 4 Discussion

### 4.1 Widespread phylogeographical breaks in the marine flora of northern Chile: ancient vicariant origin maintained by upwelling regimes

Based on oceanographic patterns and the distributional limit of marine species, two biogeographical barriers, located at 30–33°S and 42°S, have been detected along the Chilean coast (Camus, 2001; Thiel et al., 2007). In the case of seaweeds, an important biogeographic break has been observed at 42°S for brown, green, and red seaweeds, while the northern biogeographical barrier is more taxa dependent, and was observed at 30° S for brown seaweeds, and between 32°S and 33°S for red seaweeds (Santelices, 1980; Meneses and Santelices, 2000). Our result showed the existence of deep phylogeographic breaks for *Mazzaella* at 33°S and *Ahnfeltiopsis* at 30°S and 33°S. Indeed, at 33°S, we observed the separation between the putative genetic species *M. laminarioides* North and *M. laminarioides* Center, and *Ah.* sp. 2 and *Ah. vermicularis*. The species *Ah.* sp. 1 and *Ah.* sp. 2 were located on both side of the 30°S biogeographic barrier. The divergence between *M. laminarioides* North and *M. laminarioides* Center was estimated to have occurred around 7 Ma (CI: 13–2 Ma) and the divergence between *Ah.* sp. 2 and *Ah. vermicularis* around 7 Ma (CI: 13–1 Ma) while *Ah.* sp. 1 spitted from and *Ah.* sp. 2/*Ah. vermicularis* some 12 Ma (CI: 20–5 Ma). These estimations support the hypothesis that an old vicariant event could have strongly structured the genetic diversity of marine organisms at 30–33°S (Tellier et al., 2009; Haye et al., 2014; Guillemin et al., 2016). Distinct sister species or highly genetically divergent lineages separated by the 30°S biogeographic barrier have been observed in intertidal kelps of the genus *Lessonia* (*L. berteroana* and *L. spicata*: González et al., 2012; Tellier et al., 2009), and marine invertebrates with direct development (*Acanthina monodon*: Sánchez et al., 2011; *Crepipatella dilatata*; Brante et al., 2012; *Excirolana braziliensis*; Varela and Haye, 2012; *Orchestoidea tuberculata*, *Scurria scurra*, and *Tegula atra*; Haye et al., 2014), or pelagic larvae (*Stichaster striatus*: Haye et al., 2014; *Notochthamalus scabrosus*; Zakas et al., 2009). Palaeoceanographic reconstructions have inferred important changes in the surface temperature (SST) and ocean circulation in northern Chile during the Neogene and the Quaternary (Tsuchi, 2002; Kaiser et al., 2005; Villafaña and Rivadeneira, 2018). For example, a deep impact on population demography of marine organisms (Cárdenas et al., 2008) have been associated with of the decrease of SST and the activation of the upwelling systems along the coast of northern Chile and Peru that took place during mid Miocene (∼15–12 Ma) (Tsuchi, 2002). Additionally, it has been proposed that the Quaternary glacial/interglacial cycles that begun at the end of the Plio-Pleistocene transition (i.e., some 2.5 Ma) were associated with oscillations in the position of the West Wind Drift (WWD) and main ocean currents, with the WWD reaching the Chilean coast around 33°S during glacial periods (Mohtadi and Hebbeln, 2004; Kaiser et al., 2005; Mohtadi et al., 2008). These historical changes in oceanographic features could potentially be at the origin of strong historical barriers to gene flow along the northern part of the Chilean coast. In various studies, it has been suggested that the phylogeographic breaks observed at 30–33°S have been maintained up to the present time in species with low dispersal capacity by somewhat more porous barriers to gene flow linked with changes in the currents, wind, and sea surface temperature patterns in this area (Tellier et al., 2009; Sánchez et al., 2011; Montecinos et al., 2012). Currently, the 30–33°S region is characterized by latitudinal change in the type of upwellings that module the dynamics of intertidal communities of northern Chile, with strong and seasonal upwellings south of this area and more permanent but weak upwellings in the northern part of the coast (Broitman et al., 2001; Navarrete et al., 2005; Tapia et al., 2014). Changes in coastline topography (i.e., headlands) are observed at both 30°S and 33°S and were associated with the existence of strong upwelling centers at these latitudes (Figueroa and Moffat, 2000; Aiken et al., 2008). These upwelling centers have been proposed as important dispersal barriers for marine invertebrate taxa due to offshore displacement of propagules and abrupt change in chemical structure of the water column, as the oxygen concentration, for example, (Navarrete et al., 2005; Lara et al., 2019).

### 4.2 Shallow and taxa dependent genetic sub-structuring patterns in central-southern Chile: potential effect of habitat discontinuities and stochastic events

Contrasting from the 30°S and the 33°S in northern Chile, the genetic breaks observed between 33°S and 42°S generally correspond to shallow intraspecific divergence and their locations vary greatly among the marine species studied in this area (locations of genetic divergence detected in red algae: 34°S, 36°S-37°S, 39°S, and 40°S; Cid-Alda et al., 2023; Lindstrom et al., 2015; Montecinos et al., 2012; present study; brown algae: 36°S-37°S Fraser et al., 2010; and marine invertebrates: 34°S, 35°S, 36°S-37°S, 39°S and 40°S; Ewers-Saucedo et al., 2016; Guinez et al., 2016; Lara et al., 2019; Peluso et al., 2023; Quesada-Calderon et al., 2021; Sáenz-Agudelo et al., 2022). The nearly linear central-southern coastline, largely dominated by rocky shores, is interrupted by extensive sandy beaches in this region, as, for example, from Punta Morguilla to Quidico (37°43’S-38°14'S; 62 km long beach) or from Loberia to Nigue (38°39’S-39°17'S; 73 km long beach) (Thiel et al., 2007). Additionally, the coastline is interrupted by the freshwater discharge of large rivers originating in the Andes Range south of 33°S such as the Maule (35°18'S), Itata (36°23'S), Bio Bio (36°49'S), Imperial (38°47'S), and Valdivia (39°5’S) rivers (Saldías et al., 2012). Both sandy beaches and river mouths, correspond to important barriers to gene flow for marine species (Lara et al., 2019), particularly for sessile organisms presenting low dispersal capacity, as seaweeds (Fraser et al., 2010; Montecinos et al., 2012; Cid-Alda et al., 2023).

We did not find any congruence in time (i.e., numbers of fixed mutations) nor space among the phylogeographic breaks or patterns of genetic sub-structure observed in *Mazzaella*, *Asterfilopsis*, and *Ahnfeltiopsis*. A deep phylogeographic break that occurred a few million years ago (i.e., 3 Ma, CI: 7–0 Ma) was observed between *M. laminarioides* Center and *M. laminarioides* South at 39°S. Contrastingly, only much more recent intraspecific sub-structuration patterns were detected within *M. membranacea*, *Ah. vermicularis*, and *As. disciplinalis* located at 36°S, 39°S, and 40°S, respectively*.* Depending on the species, moderate to high genetic differentiation among genetic clusters (0.78 < mean Φ_ST_ between genetic groups <0.95) were observed within *M. membranacea, As. disciplinalis*, and *Ah. vermicularis*. These sub-structuring patterns do not support the existence of a unique vicariant event driving the genetic structure of marine organisms in central-southern Chile but suggest more species-specific evolutionary history.

Since the late Pliocene, the part of the Chilean coast located between 37°S and 41°S has been affected by seismic activity causing constant and extensive coastline uplifts (Folguera et al., 2011) and sediment rerouting thought the Andean drainage systems (Rehak et al., 2008). Consequently, the central-southern Chilean coastline present a high level of habitat heterogeneity, very dynamic over time. In red algae, characterized generally by poor dispersive ability and small effective population sizes (Faugeron et al., 2001; Engel et al., 2004; Cid-Alda et al., 2023), dynamic coastal and oceanographic features as the ones described between 37°S and 41°S can lead to patchy distribution, with isolated populations rapidly diverging due to genetic drift (Irwin et al., 2001; Irwin, 2002; Kuo and Avise, 2005). Large-scale mortality of intertidal and subtidal communities as a result of tectonically uplifted shoreline has been widely recorded in central-southern Chile (Castilla, 1988; Castilla et al., 2010; Jaramillo et al., 2012). Local/regional extirpations followed by recolonizations from sources populations not necessarily located nearby can lead to genetic anomalies that present the same signature as classical phylogeographic breaks but are not linked to the existence of any barrier to gene flow (Parvizi et al., 2019; Vaux et al., 2021; Parvizi et al., 2022; Vaux et al., 2022; Vaux et al., 2023). In Chile, it has been shown that regional disturbance events can rapidly reshape the pattern of distribution of genetic diversity in coastal species (Brante et al., 2019; Becheler et al., 2020). It is possible that short-term mesoscale disturbances, as the ones occurring between 37°S and 41°S in Chile due to near-shore fault rupture events causing coastal uplift (Folguera et al., 2011), could have contributed to the generation of the specific genetic sub-structuring patterns observed in the four red algae under study.

## Data Availability

The datasets presented in this study can be found in online repositories. The names of the repository/repositories and accession number(s) can be found in the article/Supplementary Material.
