# Supplementary material for: Impact of persistent barrier to gene flow and catastrophic events on red algae evolutionary history along the Chilean coast
Source: Front Genet. 2024 Mar 8;15:1336427. doi: 10.3389/fgene.2024.1336427 (PMC10957783; doi:10.3389/fgene.2024.1336427)
Supplement: Supplementary file 3 [file Table1.doc]

**Supplementary Table S1.**

|  |  | *M. laminarioides* North | | | | | | *M. laminarioides* Center | | | | | |
| --- | --- | --- | --- | --- | --- | --- | --- | --- | --- | --- | --- | --- | --- |
| Site  (code) | Position | N | nH | H^a^ | π (.10^-2^) ^a^ | H_priv_ | S | N | nH | H^a^ | π (.10^-2^) ^a^ | H_priv_ | S |
| Los Burros  (LBR) | 28°55´S, 71°31´W | 10* | 1 | 0 | 0 | 1 | 0 | - | - | - | - | - | - |
| Puerto Oscuro  (POS) | 31°24´S, 71°36´W | 19* | 1 | 0 | 0 | 1 | 0 | - | - | - | - | - | - |
| Maitencillo  (MAI) | 32°37´S, 71°38´W | 23* | 2 | 0.17 (0.10) | 0.03 (0.04) | 2 | 1 | - | - | - | - | - | - |
| Matanza  (MAT) | 34°05´S, 71°58´W | - | - | - | - | - | - | 20* | 3 | 0.66 (0.07) | 0.14 (0.12) | 1 | 3 |
| Pichilemu  (PMU) | 34°23´S, 72°01´W | - | - | - | - | - | - | 23* | 4 | 0.45 (0.12) | 0.24 (0.17) | 1 | 5 |
| Constitución  (CON) | 35°19´S, 72°26´W | - | - | - | - | - | - | 20* | 1 | 0 | 0 | 0 | 0 |
| Concepcion  (CNC) | 36°31´S, 72°57´W | - | - | - | - | - | - | 18* | 2 | 0.41 (0.10) | 0.07 (0.08) | 1 | 1 |
| Tirua  (TIR) | 37°38´S, 73°79´W | - | - | - | - | - | - | 23* | 2 | 0.30 (0.11) | 0.16 (0.13) | 0 | 3 |
| Loberia  (LOB) | 38°39´S, 73°29´W | - | - | - | - | - | - | - | - | - | - | - | - |
| Nigue  (NIG) | 39°17´S, 73°13´W | - | - | - | - | - | - | - | - | - | - | - | - |
| Pilolcura  (PIL) | 39°40´S, 73°21´W | - | - | - | - | - | - | - | - | - | - | - | - |
| Pucatrihue  (PUC) | 40°32´S, 73°43´W | - | - | - | - | - | - | - | - | - | - | - | - |
| Chiloe  (CHI) | 41°52´S, 71°01´W | - | - | - | - | - | - | - | - | - | - | - | - |
| **Total** |  | **52** | **4** | **0.68**  **(0.03)** | **1.03**  **(0.03)** | **4** | **13** | **104** | **6** | **0.61**  **(0.05)** | **0.30**  **(0.03)** | **3** | **7** |

**Supplementary Table S1.** continued.

|  |  | *M. laminarioides* South | | | | | | *M. membranacea* | | | | | |
| --- | --- | --- | --- | --- | --- | --- | --- | --- | --- | --- | --- | --- | --- |
| Site  (code) | Position | N | nH | H^a^ | π (.10^-2^) ^a^ | H_priv_ | S | N | nH | H^a^ | π (.10^-2^) ^a^ | H_priv_ | S |
| Los Burros  (LBR) | 28°55´S, 71°31´W | - | - | - | - | - | - | - | - | - | - | - | - |
| Puerto Oscuro  (POS) | 31°24´S, 71°36´W | - | - | - | - | - | - | 15 | 2 | 0.13 (0.11) | 0.02 (0.02) | 0 | 1 |
| Maitencillo  (MAI) | 32°37´S, 71°38´W | - | - | - | - | - | - | - | - | - | - | - | - |
| Matanza  (MAT) | 34°05´S, 71°58´W | - | - | - | - | - | 3 | 13 | 4 | 0.68 (0.09) | 0.15 (0.03) | 1 | 3 |
| Pichilemu  (PMU) | 34°23´S, 72°01´W | - | - | - | - | - | 5 | 14 | 3 | 0.58 (0.09) | 0.11 (0.02) | 0 | 2 |
| Constitucion  (CON) | 35°19´S, 72°26´W | - | - | - | - | - | 0 | 13 | 5 | 0.76 (0.10) | 0.17 (0.04) | 3 | 4 |
| Concepción  (CNC) | 36°31´S, 72°57´W | - | - | - | - | - | 1 | 18 | 5 | 0.75 (0.06) | 0.39 (0.04) | 2 | 5 |
| Tirua  (TIR) | 37°38´S, 73°79´W | - | - | - | - | - | 3 | 7 | 3 | 0.52 (0.21) | 0.25 (0.12) | 2 | 5 |
| Loberia  (LOB) | 38°39´S, 73°29´W | 16 | 1 | 0 | 0 | 0 | 0 | 15 | 3 | 0.26 (0.14) | 0.05 (0.03) | 2 | 2 |
| Nigue  (NIG) | 39°17´S, 73°13´W | 17 | 3 | 0.52 (0.10) | 0.98 (2.30) | 2 | 2 | 14 | 3 | 0.62 (0.10) | 0.12 (0.03) | 1 | 2 |
| Pilolcura  (PIL) | 39°40´S, 73°21´W | 21* | 4 | 0.76 (0.05) | 0.20 (0.15) | 3 | 3 | 13 | 2 | 0.28 (0.14) | 0.05 (0.03) | 0 | 1 |
| Pucatrihue  (PUC) | 40°32´S, 73°43´W | 20* | 3 | 0.56 (0.06) | 0.11 (0.10) | 2 | 2 | 10 | 1 | 0 | 0 | 0 | 0 |
| Chiloe  (CHI) | 41°52´S, 71°01´W | 20* | 3 | 0.60 (0.07) | 0.12 (0.10) | 2 | 2 | 11 | 1 | 0 | 0 | 0 | 0 |
| Total |  | **94** | **10** | **0.73**  **(0.02)** | **0.21**  **(0.02)** | **9** | **9** | **143** | **17** | **0.78 (0.03)** | **0.40 (0.02)** | **11** | **19** |

**Supplementary Table S1.** continued.

|  |  | *M.* sp. 1 | | | | | | *As. disciplinalis* | | | | | |
| --- | --- | --- | --- | --- | --- | --- | --- | --- | --- | --- | --- | --- | --- |
| Site  (code) | Position | N | nH | H^a^ | π (.10^-2^) ^a^ | H_priv_ | S | N | nH | H^a^ | π (.10^-2^) ^a^ | H_priv_ | S |
| Los Burros  (LBR) | 28°55´S, 71°31´W | - | - | - | - | - | - | - | - | - | - | - | - |
| Puerto Oscuro  (POS) | 31°24´S, 71°36´W | - | - | - | - | - | - | - | - | - | - | - | - |
| Maitencillo  (MAI) | 32°37´S, 71°38´W | 5 | 3 | 0.70  (0.21) | 0.14  (0.05) | 2 | 2 | - | - | - | - | - | - |
| Matanza  (MAT) | 34°05´S, 71°58´W | - | - | - | - | - | - | 15 | 2 | 0.13 (0.11) | 0.02 (0.02) | 0 | 1 |
| Pichilemu  (PMU) | 34°23´S, 72°01´W | - | - | - | - | - | - | 16 | 4 | 0.44 (0.15) | 0.08 (0.03) | 2 | 3 |
| Constitución  (CON) | 35°19´S, 72°26´W | - | - | - | - | - | - | 16 | 3 | 0.54 (0.10) | 0.10 (0.02) | 1 | 2 |
| Concepcion  (CNC) | 36°31´S, 72°57´W | - | - | - | - | - | - | 17 | 1 | 0 | 0 | 0 | 0 |
| Tirua  (TIR) | 37°38´S, 73°79´W | 4 | 1 | 0 | 0 | 0 | 0 | 15 | 3 | 0.26 (0.14) | 0.05 (0.03) | 1 | 2 |
| Loberia  (LOB) | 38°39´S, 73°29´W | - | - | - | - | - | - | 3 | 1 | 0 | 0 | 0 | 0 |
| Nigue  (NIG) | 39°17´S, 73°13´W | - | - | - | - | - | - | 15 | 3 | 0.26 (0.14) | 0.05 (0.03) | 1 | 2 |
| Pilolcura  (PIL) | 39°40´S, 73°21´W | - | - | - | - | - | - | 12 | 2 | 0.30 (0.15) | 0.05 (0.03) | 1 | 1 |
| Pucatrihue  (PUC) | 40°32´S, 73°43´W | - | - | - | - | - | - | 15 | 1 | 0 | 0 | 0 | 0 |
| Chiloe  (CHI) | 41°52´S, 71°01´W | - | - | - | - | - | - | 16 | 2 | 0.46 (0.10) | 0.08 (0.02) | 1 | 1 |
| Total |  | **9** | **3** | **0.41**  **(0.03)** | **0.08**  **(0.04)** | **2** | **2** | **140** | **11** | **0.56 (0.04)** | **0.36 (0.04)** | **7** | **13** |

**Supplementary Table S1.** continued.

|  |  | *Ah. vermicularis* | | | | | | *Ah.* sp. 2 | | | | | |
| --- | --- | --- | --- | --- | --- | --- | --- | --- | --- | --- | --- | --- | --- |
| Site  (code) | Position | N | nH | H^a^ | π (.10^-2^) ^a^ | H_priv_ | S | N | nH | H^a^ | π (.10^-2^) ^a^ | H_priv_ | S |
| Los Burros  (LBR) | 28°55´S, 71°31´W | - | - | - | - | - | - | - | - | - | - | - | - |
| Puerto Oscuro  (POS) | 31°24´S, 71°36´W | - | - | - | - | - | - | 19 | 3 | 0.29  (0.13) | 0.07 (0.03) | 1 | 2 |
| Maitencillo  (MAI) | 32°37´S, 71°38´W | - | - | - | - | - | - | 17 | 6 | 0.82 (0.06) | 0.30 (0.03) | 4 | 4 |
| Matanza  (MAT) | 34°05´S, 71°58´W | 15 | 1 | 0 | 0 | 0 | 0 | - | - | - | - | - | - |
| Pichilemu  (PMU) | 34°23´S, 72°01´W | 11 | 1 | 0 | 0 | 0 | 0 | - | - | - | - | - | - |
| Constitución  (CON) | 35°19´S, 72°26´W | 14 | 2 | 0.14 (0.12) | 0.05 (0.04) | 0 | 2 | - | - | - | - | - | - |
| Concepción  (CNC) | 36°31´S, 72°57´W | 14 | 2 | 0.14 (0.12) | 0.03 (0.02) | 1 | 1 | - | - | - | - | - | - |
| Tirua  (TIR) | 37°38´S, 73°79´W | 15 | 3 | 0.26 (0.14) | 0.14 (0.95) | 2 | 5 | - | - | - | - | - | - |
| Loberia  (LOB) | 38°39´S, 73°29´W | 31 | 1 | 0 | 0 | 0 | 0 | - | - | - | - | - | - |
| Nigue  (NIG) | 39°17´S, 73°13´W | 13 | 4 | 0.76 (0.07) | 0.64 (0.07) | 1 | 7 | - | - | - | - | - | - |
| Pilolcura  (PIL) | 39°40´S, 73°21´W | 15 | 2 | 0.34 (0.13) | 0.06 (0.02) | 0 | 1 | - | - | - | - | - | - |
| Pucatrihue  (PUC) | 40°32´S, 73°43´W | 14 | 4 | 0.40 (0.16) | 0.11 (0.05) | 3 | 3 | - | - | - | - | - | - |
| Chiloé  (CHI) | 41°52´S, 71°01´W | 13 | 2 | 0.51 (0.08) | 0.89 (0.01) | 1 | 1 | - | - | - | - | - | - |
| Total |  | 155 | 12 | 0.75  (0.02) | 0.60  (0.03) | 8 | 11 | 36 | 7 | 0.61 (0.09) | 0.22 (0.04) | 5 | 5 |
