# Supplementary material for: Impact of persistent barrier to gene flow and catastrophic events on red algae evolutionary history along the Chilean coast
Source: Front Genet. 2024 Mar 8;15:1336427. doi: 10.3389/fgene.2024.1336427 (PMC10957783; doi:10.3389/fgene.2024.1336427)
Supplement: Supplementary file 4 [file Table4.docx]

**Supplementary Table S4.**

| ***M. laminarioides* North** (Φ_ST_ below the diagonal - p value above the diagonal) | | | | | | | | | | | | | |
| --- | --- | --- | --- | --- | --- | --- | --- | --- | --- | --- | --- | --- | --- |
|  | LBR | POS | MAI | MAT | PMU | CON | CNC | TIR | LOB | NIG | PIL | PUC | CHI |
| LBR | * | 0.000 | 0.000 | - | - | - | - | - | - | - | - | - | - |
| POS | 1.000 | * | 0.000 | - | - | - | - | - | - | - | - | - | - |
| MAI | 0.988 | 0.990 | * | - | - | - | - | - | - | - | - | - | - |
| MAT | 0.985 | 0.989 | 0.988 | * | - | - | - | - | - | - | - | - | - |
| PMU | 0.974 | 0.980 | 0.979 | - | * | - | - | - | - | - | - | - | - |
| CON | 1.000 | 1.000 | 0.997 | - | - | * | - | - | - | - | - | - | - |
| CNC | 0.992 | 0.994 | 0.992 | - | - | - | * | - | - | - | - | - | - |
| TIR | 0.983 | 0.987 | 0.986 | - | - | - | - | * | - | - | - | - | - |
| LOB | 1.000 | 1.000 | 0.997 | - | - | - | - | - | * | - | - | - | - |
| NIG | 0.990 | 0.992 | 0.991 | - | - | - | - | - | - | * | - | - | - |
| PIL | 0.978 | 0.983 | 0.982 | - | - | - | - | - | - | - | * | - | - |
| PUC | 0.988 | 0.991 | 0.989 | - | - | - | - | - | - | - | - | * | - |
| CHI | 0.987 | 0.990 | 0.989 | - | - | - | - | - | - | - | - | - | * |
| ***M. laminarioides* Center** (Φ_ST_ below the diagonal - p value above the diagonal) | | | | | | | | | | | | | |
|  | LBR | POS | MAI | MAT | PMU | CON | CNC | TIR | LOB | NIG | PIL | PUC | CHI |
| LBR | * | - | - | 0.000 | 0.000 | 0.000 | 0.000 | 0.000 | - | - | - | - | - |
| POS | - | * | - | 0.000 | 0.000 | 0.000 | 0.000 | 0.000 | - | - | - | - | - |
| MAI | - | - | * | 0.000 | 0.000 | 0.000 | 0.000 | 0.000 | - | - | - | - | - |
| MAT | - | - | - | * | 0.000 | 0.000 | 0.000 | 0.000 | - | - | - | - | - |
| PMU | - | - | - | 0.626 | * | 0.049 | 0.000 | 0.000 | - | - | - | - | - |
| CON | - | - | - | 0.887 | 0.144 | * | 0.000 | 0.550 | - | - | - | - | - |
| CNC | - | - | - | 0.854 | 0.395 | 0.718 | * | 0.110 | - | - | - | - | - |
| TIR | - | - | - | 0.715 | 0.000 | 0.123 | 0.454 | * | - | - | - | - | - |
| LOB | - | - | - | 0.971 | 0.953 | 1.000 | 0.987 | 0.969 | * | - | - | - | - |
| NIG | - | - | - | 0.957 | 0.943 | 0.986 | 0.973 | 0.958 | - | * | - | - | - |
| PIL | - | - | - | 0.943 | 0.933 | 0.970 | 0.958 | 0.946 | - | - | * | - | - |
| PUC | - | - | - | 0.956 | 0.942 | 0.983 | 0.971 | 0.957 | - | - | - | * | - |
| CHI | - | - | - | 0.954 | 0.941 | 0.981 | 0.970 | 0.955 | - | - | - | - | * |
| ***M. laminarioides* South** (Φ_ST_ below the diagonal - p value above the diagonal) | | | | | | | | | | | | | |
|  | LBR | POS | MAI | MAT | PMU | CON | CNC | TIR | LOB | NIG | PIL | PUC | CHI |
| LBR | * | - | - | - | - | - | - | - | 0.000 | 0.000 | 0.000 | 0.000 | 0.000 |
| POS | - | * | - | - | - | - | - | - | 0.000 | 0.000 | 0.000 | 0.000 | 0.000 |
| MAI | - | - | * | - | - | - | - | - | 0.000 | 0.000 | 0.000 | 0.000 | 0.000 |
| MAT | - | - | - | * | - | - | - | - | 0.000 | 0.000 | 0.000 | 0.000 | 0.000 |
| PMU | - | - | - | - | * | - | - | - | 0.000 | 0.000 | 0.000 | 0.000 | 0.000 |
| CON | - | - | - | - | - | * | - | - | 0.000 | 0.000 | 0.000 | 0.000 | 0.000 |
| CNC | - | - | - | - | - | - | * | - | 0.000 | 0.000 | 0.000 | 0.000 | 0.000 |
| TIR | - | - | - | - | - | - | - | * | 0.000 | 0.000 | 0.000 | 0.000 | 0.000 |
| LOB | - | - | - | - | - | - | - | - | * | 0.000 | 0.000 | 0.002 | 0.005 |
| NIG | - | - | - | - | - | - | - | - | 0.626 | * | 0.000 | 0.000 | 0.000 |
| PIL | - | - | - | - | - | - | - | - | 0.581 | 0.613 | * | 0.000 | 0.000 |
| PUC | - | - | - | - | - | - | - | - | 0.297 | 0.519 | 0.546 | * | 0.000 |
| CHI | - | - | - | - | - | - | - | - | 0.229 | 0.492 | 0.530 | 0.292 | * |
| ***M. membranacea*** (Φ_ST_ below the diagonal - p value above the diagonal) | | | | | | | | | | | | | |
|  | LBR | POS | MAI | MAT | PMU | CON | CNC | TIR | LOB | NIG | PIL | PUC | CHI |
| LBR | * | - | - | - | - | - | - | - | - | - | - | - | - |
| POS | - | * | - | 0.000 | 0.000 | 0.000 | 0.000 | 0.000 | 0.000 | 0.000 | 0.000 | 0.000 | 0.000 |
| MAI | - | - | * | - | - | - | - | - | - | - | - | - | - |
| MAT | - | 0.820 | - | * | 0.014 | 0.279 | 0.015 | 0.000 | 0.000 | 0.000 | 0.000 | 0.000 | 0.000 |
| PMU | - | 0.887 | - | 0.197 | * | 0.013 | 0.001 | 0.000 | 0.000 | 0.000 | 0.000 | 0.000 | 0.000 |
| CON | - | 0.832 | - | 0.032 | 0.139 | * | 0.003 | 0.000 | 0.000 | 0.000 | 0.000 | 0.000 | 0.000 |
| CNC | - | 0.405 | - | 0.194 | 0.354 | 0.259 | * | 0.000 | 0.000 | 0.000 | 0.000 | 0.000 | 0.000 |
| TIR | - | 0.637 | - | 0.750 | 0.818 | 0.762 | 0.477 | * | 0.124 | 0.056 | 0.103 | 0.171 | 0.117 |
| LOB | - | 0.812 | - | 0.853 | 0.899 | 0.858 | 0.582 | 0.070 | * | 0.007 | 0.208 | 0.999 | 0.999 |
| NIG | - | 0.698 | - | 0.805 | 0.857 | 0.814 | 0.554 | 0.100 | 0.140 | * | 0.409 | 0.049 | 0.060 |
| PIL | - | 0.813 | - | 0.847 | 0.895 | 0.851 | 0.568 | 0.073 | 0.045 | 0.001 | * | 0.493 | 0.468 |
| PUC | - | 0.914 | - | 0.865 | 0.913 | 0.865 | 0.557 | 0.054 | 0.000 | 0.137 | 0.055 | * | 0.999 |
| CHI | - | 0.917 | - | 0.870 | 0.916 | 0.870 | 0.567 | 0.068 | 0.000 | 0.149 | 0.065 | 0.000 | * |
| ***Asterfilopsis disciplinalis*** (Φ_ST_ below the diagonal - p value above the diagonal) | | | | | | | | | | | | | |
|  | LBR | POS | MAI | MAT | PMU | CON | CNC | TIR | LOB | NIG | PIL | PUC | CHI |
| LBR | * | - | - | - | - | - | - | - | - | - | - | - | - |
| POS | - | * | - | - | - | - | - | - | - | - | - | - | - |
| MAI | - | - | * | - | - | - | - | - | - | - | - | - | - |
| MAT | - | - | - | * | 0.999 | 0.172 | 0.455 | 0.999 | 0.000 | 0.999 | 0.215 | 0.000 | 0.000 |
| PMU | - | - | - | 0.000 | * | 0.391 | 0.033 | 0.415 | 0.001 | 0.399 | 0.149 | 0.000 | 0.000 |
| CON | - | - | - | 0.100 | 0.024 | * | 0.005 | 0.033 | 0.000 | 0.040 | 0.016 | 0.000 | 0.000 |
| CNC | - | - | - | 0.008 | 0.038 | 0.230 | * | 0.221 | 0.001 | 0.208 | 0.181 | 0.000 | 0.000 |
| TIR | - | - | - | 0.000 | 0.020 | 0.161 | 0.008 | * | 0.000 | 0.999 | 0.166 | 0.000 | 0.000 |
| LOB | - | - | - | 0.893 | 0.687 | 0.660 | 1.000 | 0.780 | * | 0.001 | 0.003 | 0.000 | 0.001 |
| NIG | - | - | - | 0.000 | 0.020 | 0.161 | 0.008 | 0.000 | 0.780 | * | 0.185 | 0.000 | 0.000 |
| PIL | - | - | - | 0.074 | 0.050 | 0.170 | 0.133 | 0.051 | 0.791 | 0.051 | * | 0.000 | 0.000 |
| PUC | - | - | - | 0.986 | 0.952 | 0.943 | 1.000 | 0.973 | 1.000 | 0.973 | 0.974 | * | 0.058 |
| CHI | - | - | - | 0.944 | 0.915 | 0.908 | 0.958 | 0.931 | 0.908 | 0.931 | 0.928 | 0.257 | * |
| ***Ah. vermicularis*** (Φ_ST_ below the diagonal - p value above the diagonal) | | | | | | | | | | | | | |
|  | LBR | POS | MAI | MAT | PMU | CON | CNC | TIR | LOB | NIG | PIL | PUC | CHI |
| LBR | * | - | - | - | - | - | - | - | - | - | - | - | - |
| POS | - | * | - | - | - | - | - | - | - | - | - | - | - |
| MAI | - | - | * | - | - | - | - | - | - | - | - | - | - |
| MAT | - | - | - | * | 0.999 | 0.494 | 0.468 | 0.000 | 0.000 | 0.000 | 0.000 | 0.000 | 0.000 |
| PMU | - | - | - | 0.000 | * | 0.999 | 0.999 | 0.000 | 0.000 | 0.000 | 0.000 | 0.000 | 0.000 |
| CON | - | - | - | 0.005 | 0.000 | * | 0.999 | 0.000 | 0.000 | 0.000 | 0.000 | 0.000 | 0.000 |
| CNC | - | - | - | 0.005 | 0.000 | 0.000 | * | 0.000 | 0.000 | 0.000 | 0.000 | 0.000 | 0.000 |
| TIR | - | - | - | 0.816 | 0.791 | 0.731 | 0.773 | * | 0.101 | 0.002 | 0.000 | 0.000 | 0.000 |
| LOB | - | - | - | 1.000 | 1.000 | 0.952 | 0.977 | 0.085 | * | 0.000 | 0.000 | 0.000 | 0.000 |
| NIG | - | - | - | 0.611 | 0.566 | 0.561 | 0.585 | 0.394 | 0.641 | * | 0.005 | 0.000 | 0.000 |
| PIL | - | - | - | 0.972 | 0.967 | 0.949 | 0.960 | 0.903 | 0.982 | 0.359 | * | 0.000 | 0.000 |
| PUC | - | - | - | 0.954 | 0.946 | 0.931 | 0.942 | 0.889 | 0.979 | 0.444 | 0.563 | * | 0.000 |
| CHI | - | - | - | 0.964 | 0.957 | 0.940 | 0.952 | 0.894 | 0.977 | 0.411 | 0.479 | 0.622 | * |
| ***Ah.* sp. 2** (Φ_ST_ below the diagonal - p value above the diagonal) | | | | | | | | | | | | | |
|  | LBR | POS | MAI | MAT | PMU | CON | CNC | TIR | LOB | NIG | PIL | PUC | CHI |
| LBR | * | - | - | - | - | - | - | - | - | - | - | - | - |
| POS | - | * | 0.000 | - | - | - | - | - | - | - | - | - | - |
| MAI | - | 0.340 | * | - | - | - | - | - | - | - | - | - | - |
| MAT | - | - | - | * | - | - | - | - | - | - | - | - | - |
| PMU | - | - | - | - | * | - | - | - | - | - | - | - | - |
| CON | - | - | - | - | - | * | - | - | - | - | - | - | - |
| CNC | - | - | - | - | - | - | * | - | - | - | - | - | - |
| TIR | - | - | - | - | - | - | - | * | - | - | - | - | - |
| LOB | - | - | - | - | - | - | - | - | * | - | - | - | - |
| NIG | - | - | - | - | - | - | - | - | - | * | - | - | - |
| PIL | - | - | - | - | - | - | - | - | - | - | * | - | - |
| PUC | - | - | - | - | - | - | - | - | - | - | - | * | - |
| CHI | - | - | - | - | - | - | - | - | - | - | - | - | * |
