# Supplementary material for: Impact of persistent barrier to gene flow and catastrophic events on red algae evolutionary history along the Chilean coast
Source: Front Genet. 2024 Mar 8;15:1336427. doi: 10.3389/fgene.2024.1336427 (PMC10957783; doi:10.3389/fgene.2024.1336427)
Supplement: Supplementary file 5 [file DataSheet1.docx]

Supplementary Material

**Supplementary Table S1.** Location of sampling sites and indices of genetic diversity calculated using the COI sequences of *M. laminarioides* North, *M. laminarioides* Center, *M. laminarioides* South, *M. membranacea*, *M.* sp.1, *As. disciplinalis*, *Ah. vermicularis*, and *Ah.* sp. 2 for each population sampled across Chilean coast. For each site, the abbreviation (code) and the geographic coordinates are indicated. N: total number of sequences with the number of sequences added for *M. laminarioides* from Montecinos et al. (2012) indicated with an asterisk (*); nH: number of haplotypes; H: gene diversity; π: nucleotide diversity; S: number of polymorphic sites; Hpriv: number of private haplotypes; -: not estimated. ^a^Standard deviations are in brackets.

**Supplementary Table S2.** GenBank accession numbers of COI and *rbc*L sequences used in *Mazzaella*, *Asterfilopsis*, and *Ahnfeltiopsis* phylogenetic reconstruction. GenBank accession numbers of sequences obtained in our study are in bold. * = sequences used for estimation of time of divergence; highlighted in grey = sequences used as outgroup.

**Supplementary Table S3.** Geographical distribution of COI haplotypes used in *Mazzaella*, *Asterfilopsis* and *Ahnfeltiopsis* phylogeographic structure analysis. Sample site name and codes are as in Table 1.

**Supplementary Table S4.** Estimates of pairwise Φ_ST_ (below the diagonal) and *p* value (above the diagonal) calculated from the COI data set. Values are given for seven putative genetic species: *Mazzaella laminarioides* North, *M. laminarioides* Center, *M. laminarioides* South, *M. membranacea*, *Asterfilopsis disciplinalis, Ahnfeltiopsis vermicularis*, and *Ah.* sp. 2

**
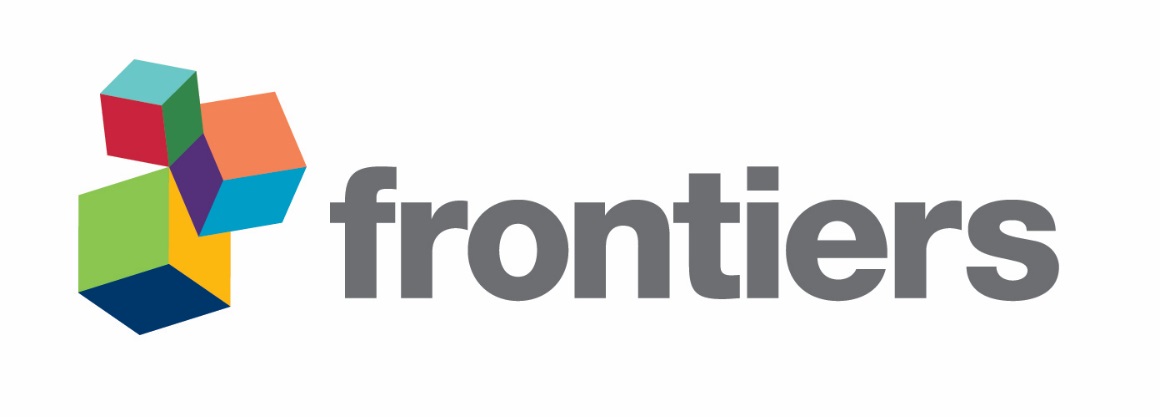
**
